# New-Onset Psychosis Associated With Acute Cerebral Hemorrhage: A Case Report

**DOI:** 10.7759/cureus.85132

**Published:** 2025-05-31

**Authors:** Isabella Cosmello, Savraj S Sekhon, Maria A Guirguis, Sarah Tedesco, Archna Sarwal

**Affiliations:** 1 Psychiatry and Behavioral Sciences, American University of Antigua, St. John's, ATG; 2 Psychiatry and Behavioral Sciences, Richmond University Medical Center, New York, USA; 3 General Surgery, Brown University Health, Providence, USA

**Keywords:** atypical psychosis, auditory hallucinations, brain bleed, cerebral cavernoma, spontaneous cerebral hemorrhage, visual hallucination

## Abstract

Psychosis is a disorder marked by altered perceptions and thoughts, and is traditionally associated with psychiatric conditions, but its occurrence in the context of organic brain lesions remains under-explored. This case report describes a 36-year-old African American female who presented with a unique psychiatric profile, characterized by subconscious somatic symptoms and delusions, which coincided with imaging findings of a subarachnoid hemorrhage. The alignment between the psychiatric symptoms and the site of the hemorrhage is highly unusual and suggests a direct link between brain injury and psychotic manifestation. While post-stroke or lesion-related psychosis often lacks such precise lateralization, this case stands out for its diagnostic implications. The somatic delusions and hallucinations observed, particularly those associated with the left frontal lobe and insular cortex, further support a neuroanatomical contribution to the psychotic symptoms.

Although somatic delusions have been documented in patients with brain lesions, their lateralization is not well understood, and this case provides a rare opportunity to explore the role of specific brain regions in their development. To our knowledge, this may be one of the first instances in which psychotic symptoms localize ipsilaterally to a subarachnoid hemorrhage. This case highlights the complex relationship between neurobiology, neuroanatomy, and psychiatric symptoms, underscoring the need to consider organic brain lesions in the differential diagnosis of new-onset psychosis. Given the paucity of literature on this subject, this report calls for further investigation into how brain bleeds and other organic lesions contribute to psychiatric symptoms, with the aim of improving diagnostic accuracy and early intervention strategies. Understanding these associations may help clinicians refine their diagnostic approach and enhance patient care in cases where psychiatric and neurological symptoms overlap.

## Introduction

Psychosis is a condition in which severe disruptions in thinking, perception, and behavior result in a distorted sense of reality. This altered sense of reality involves hallucinations, delusions, or both that persist despite contradictory evidence. These symptoms are routinely seen exclusively through a lens of primary psychiatric illness. Yet, increasingly, it is critical to note the potential interplay between neuroanatomy and psychiatric symptoms to more accurately diagnose and manage treatment. As such, these symptoms may be the manifestation of an underlying neurologic pathology [1]. In particular, somatic delusions pose a unique diagnostic challenge when superimposed onto neurologic conditions, as they may be misinterpreted as purely a psychiatric pathology as opposed to a secondary one, delaying appropriate neurological workup and intervention. This article presents a 36-year-old African American female with a unique psychiatric presentation, featuring subconscious somatic symptoms, auditory and visual hallucinations, paranoid delusions, and depressive symptoms with unexpected neuroimaging results. The findings revealed an acute left frontotemporal subarachnoid hemorrhage ipsilateral to the presenting somatic psychiatric symptoms. Further research into the early detection of untreated aneurysms and the somatic indicators associated with them could aid in timely diagnosis and treatment. Bridging the gap between neurobiology, neuroanatomy, and psychosis could offer new perspectives on diagnosis and open up alternative diagnostic pathways for clinicians. The impact that neurobiology and neuroanatomy have on the formation of the bleed, paralleling psychotic symptomatology, remains limited [2]. This case offers a valuable opportunity to explore how brain bleeds can coincide with psychotic symptomatology, highlighting the role of neurobiology and neuroanatomy in the development of such symptoms.

## Case presentation

A 36-year-old African American female presented to the psychiatric emergency department (ED), describing a sensation, stating "something is on top of my head." She appeared slightly disheveled, with diminished psychomotor activity and generalized weakness. Her behavior was guarded, characterized by poor eye contact and impaired concentration. Speech was hesitant, with decreased rate, rhythm, and volume. Her affect was constricted, and her mood appeared depressed.

On mental status examination, the patient displayed significant poverty of thought, responding briefly only to direct questioning. Thought blocking was accompanied by somatic and paranoid delusions, as well as auditory and visual hallucinations. She described an object pressing down on her head and expressed a fixed belief in a "growth from my brain." The patient reported seeing a "big black thing" on her head when passing mirrors, and persistently hearing a man’s voice. She also stated persistent feelings that someone was after her. The patient's Positive and Negative Syndrome Scale (PANSS) score is 59 points, with the positive scale scoring 12 points and the negative scale scoring 11 points, indicating moderate symptom severity across both domains.

The patient stated a past psychiatric evaluation "a long time ago" that detected depression, but denied ongoing psychiatric treatment. However, she acknowledged prior antidepressant use, but she discontinued abruptly after auditory hallucinations instructed her to stop. The patient identified significant psychosocial stressors, notably unresolved grief from the death of her mother two years prior and the recent disappearance of her brother. She expressed feelings of overwhelming sadness and helplessness, stating, "I wish I were dead and could be with my mother," but denied suicidal intent, emphasizing her desire to care for her three children.

To gain further insight into the patient's condition, a biopsychosocial model was used. Information regarding the patient's history was obtained from the patient herself, her aunt, and a review of her electronic medical records (Table 1).

**Table 1:** Biopsychosocial Formulation. The table presents background information gathered from collateral and a thorough chart review from an electronic medical record.

	Biological	Psychological	Social
Predisposing	Family history of schizophrenia in mother, previous suicide attempt from aunt. Past medical history of anemia and auditory disability; past psychiatric history of major depressive disorder; history of mental illness since childhood.	Low self-esteem, poor coping mechanisms with the losses in her life, thoughts about death, long history of mental illness since childhood.	Poor relationships; low socioeconomic status; potential domestic violence; young parenthood; unemployed; dropped out of 12th grade due to first pregnancy; history of special education in childhood.
Precipitating	Reported abuse of nicotine products and cannabis use. Medication nonadherence.	Unable to grieve the loss of her mother; stress from relationship with the father of her children; reported substance abuse with nicotine and cannabis.	Reported auditory disability; loss of mother; interpersonal trauma from the father of her children.
Perpetuating	Reported auditory disability; reported abuse of nicotine and cannabis.	Poor relationships, a history of domestic violence, and poor coping skills.	Relationship discord; potential domestic violence; unemployed.
Protective	Easy temperament; resilience.	Ability to be reflective or modulate her affect.	Supportive family through her aunt and children.

Laboratory studies, including complete blood count, comprehensive metabolic panel, thyroid function tests, and serial assessments, returned normal results. Urine toxicology was positive for marijuana. Due to the new onset of lethargy and weakness, an internal medicine consultation was obtained. The physical examination and vital signs at this time were otherwise unremarkable.

**Figure 1:**
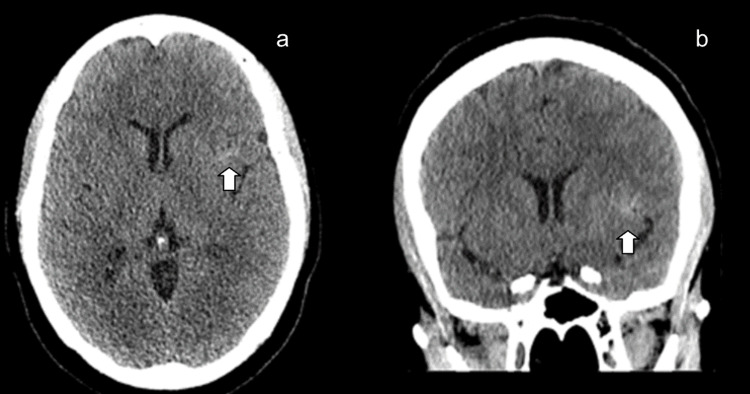
Computed Tomography (CT) of the Head Without Contrast Impression: Acute left subarachnoid hemorrhage in the left frontotemporal region without significant midline shift or mass effect (white arrow). (a) Axial view (b) Coronal view.

Initial psychiatric diagnostic impressions included severe major depressive disorder and complicated grief disorder lasting over 12 months. Given the atypical symptomatology, a computed tomography (CT) scan of the head without contrast was obtained, which demonstrated an acute left frontotemporal subarachnoid hemorrhage without significant midline shift or mass effect (Figure 1). Following these findings, the patient was promptly transferred to the intensive care unit (ICU) for close monitoring and further evaluation.

Upon arrival in the ICU, neurosurgical evaluation was conducted. The patient elaborated on her symptoms, describing a longstanding sensation of pressure atop her head exacerbated by standing, and a childhood history of recurrent headaches. She reported ongoing neurological follow-up elsewhere for "something in her brain," though she denied recent trauma. Neurological examination was unremarkable. Clinical evaluation yielded a Glasgow Coma Scale score of 15, indicative of minimal neurologic impairment, and a Hunt-Hess score of Grade I, corresponding to mild subarachnoid hemorrhage severity.

**Figure 2:**
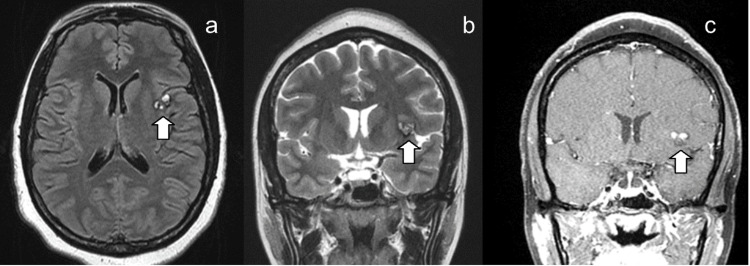
Magnetic Resonance Imaging (MRI) of the Head With/Without Contrast Impression: Area of susceptibility noted adjacent to the left insular cortex with parenchymal and subarachnoid components correlating with the hyperdensity as seen on prior CT head, consistent with hemorrhage (white arrow). (a) Axial view; (b) Coronal view; (b) Coronal view post-contrast.

During her three-day stay in the ICU, the patient underwent additional neuroimaging, including computed tomography angiography (CTA) of the head and neck, magnetic resonance imaging (MRI) of the brain, and carotid arteriography. Studies were unremarkable, and MRI showed an area of susceptibility noted adjacent to the left insular cortex with parenchymal and subarachnoid components correlating with the hyperdensity as seen on prior CT head, consistent with hemorrhage (Figure 2). Multidisciplinary care was coordinated by teams from Intensive Care Medicine, Psychiatry, Neurointerventional Radiology, Neurosurgery, and Ophthalmology. 

At the conclusion of her hospital stay, she was deemed clinically stable for discharge home. Discharge planning included arrangements for Visiting Nurse Association (VNA) services for physical therapy, which recommended the use of a rolling walker. Neurointerventional Radiology found no evidence of aneurysm, and Neurosurgery determined that surgical intervention was unnecessary. Ophthalmology and Psychiatry scheduled an outpatient follow-up, and the internal medicine team advised a follow-up appointment with her primary care physician and neurology/neurosurgery.

Prior to discharge, the treatment plan and necessary outpatient care arrangements were thoroughly reviewed with the patient and her family, ensuring clarity and continuity of care. The discharge plan, including the need for follow-up care and services, was thoroughly discussed with the patient and her next of kin (Table 2).

**Table 2:** Timeline of Clinical Events The table displays the chronological summary of the patient’s clinical course. A brief day-by-day outline of the patient’s presentation, ICU evaluation, diagnostic imaging, multidisciplinary management, and discharge planning. ED: emergency department; GCS: Glasgow Coma Scale; CTA: computed tomography angiography; VNA: Visiting Nurse Association

	Clinical Events
Day 1: Initial Presentation	The patient presented to the psychiatric ED with somatic and paranoid delusions, auditory/visual hallucinations, and symptoms of major depression and complicated grief. Mental status exam showed thought blocking and psychomotor slowing. CT head revealed an acute left frontotemporal subarachnoid hemorrhage; the patient was transferred to the ICU.
Day 2: ICU Evaluation and Imaging	GCS 15; Hunt-Hess Grade I. Neurologic exam unremarkable. The patient described longstanding head pressure and prior neurologic symptoms. Underwent head and neck CTA. Multidisciplinary consultations initiated. Psychiatrically, the patient endorses a depressed mood and anxiety. She denies current auditory or visual hallucinations, though she occasionally hears the voice of one of her children’s fathers.
Day 3: Advanced Imaging and Continued Monitoring	Underwent head MRI and carotid arteriography. Imaging confirmed left insular parenchymal and subarachnoid hemorrhage; no aneurysm detected. Psychiatry, Neurosurgery, Ophthalmology, and Neurointerventional Radiology continued evaluation. Psychiatrically, the patient endorses anxiety. She denies current auditory or visual hallucinations.
Day 4: Discharge and Outpatient Planning	The patient was deemed stable for discharge. VNA services arranged for physical therapy with a rolling walker. Outpatient follow-ups planned with Psychiatry, Ophthalmology, Neurology, and Primary Care. No surgical intervention required.

## Discussion

This case involves a 36-year-old African American female who displayed a striking combination of somatic symptoms and delusions, both localized to the same side as a brain bleed. While somatic symptoms and psychosis have been documented in neurological conditions, the unique localization of these symptoms to the side of the lesion highlights the complex interaction between neuroanatomy, neurobiology, and psychiatric symptoms.

Recent studies on the frontal lobes and neuropsychiatric disorders have emphasized the importance of frontal-subcortical circuits in driving various cognitive and behavioral symptoms. Disruption to these pathways, particularly the prefrontal cortex and the basal ganglia, has been associated with emotional and cognitive disturbances, including fixed delusions and altered thought processes [3]. The patient’s presentation was consistent with somatic delusions, hallucinations, thought blocking, poverty of speech, and a restricted affect. These features coincide closely with injury in the left dorsolateral prefrontal and left insular cortex, which was suggested by neuroimaging [4]. By accurately pinpointing these lesions, clinicians can better differentiate between primary psychiatric conditions and those with neurological origins. These findings suggest that neuroimaging may have diagnostic value in select psychiatric presentations, particularly when atypical features are present. However, further studies are needed to determine whether routine neuroimaging is warranted in similar cases. This can ultimately guide more targeted treatment approaches and improve patient outcomes. Delusions often reflect neural impairments, so understanding their neural basis is crucial. Further research should explore the pathophysiologic mechanisms consistent with these presenting symptoms.

The left insular cortex is involved in integrating internal bodily sensations with emotional salience, and its disruption has been linked to somatic delusions and tactile hallucinations. Left frontal lobe lesions have been associated with psychosis, depressive symptoms, and reduced speech output [5]. In contrast, right hemisphere damage, especially involving frontal and temporal areas, has been associated with disinhibited behaviors, mania, and emotional lability. The lateralized nature of the presenting illness indicates how emotional and cognitive dysfunction varies depending on the site of injury. While this patient’s symptoms reflect left-sided dysfunction, the presence of somatic delusions may also suggest right-hemisphere integrative processes [6]. The concept of “neurological psychosis,” often observed after stroke or brain injury, structures these symptoms as a disruption of sensory integrative and abnormal salience processing, rather than an underlying primary psychiatric pathology [7].

Psychosis following an aneurysmal subarachnoid hemorrhage or other cerebrovascular events has been documented, most commonly manifested as delirium or cognitive impairment, but less frequently as a true psychotic disorder. Antipsychotic medications are frequently used in these cases, particularly when behavioral disturbances or psychosis are present, though the precise indications and duration of treatment remain unclear. Recent research highlights the importance of being alert to neuropsychiatric symptoms post-subarachnoid hemorrhage, pointing out that unusual or localized psychiatric symptoms seen in this patient may actually have an organic cause. The duration of antipsychotic treatment should be tailored to each patient, weighing the benefits against the risks, such as potential side effects like metabolic dysregulation or extrapyramidal symptoms [8]. In the absence of clear clinical guidelines, treatment decisions are often left to the clinician's judgment. This gap in research makes it crucial to refine protocols for managing post-stroke psychosis. The way symptoms vary from patient to patient, as seen in this case, underscores the need for ongoing reassessment to ensure care is appropriately adjusted.

In the absence of standardized protocols, the duration of therapy often depends on clinical judgment. This presents a significant gap in the management of post-stroke or post-hemorrhagic psychosis. Treatment must be individualized and guided by continual reassessment, particularly when psychotic symptoms are suspected to arise from structural brain lesions. While antipsychotic medications can be effective in mitigating acute psychiatric disturbances, prolonged use requires regular evaluation to prevent iatrogenic harm and to assess the continued necessity of pharmacologic intervention [9]. The heterogeneity of symptom presentation, as seen in this case, further supports the call for optimizing clinical guidelines and longitudinal studies to inform evidence-based care.

Psychotic symptoms after brain injury can also arise from biochemical disturbances, including dysregulation of dopamine, serotonin, and glutamate systems, alongside neuroinflammation and mitochondrial dysfunction [10]. These factors may contribute to impaired neural signaling, oxidative stress, and ultimately, the emergence of psychosis. Involvement of the parietal, temporal, and frontal lobes can cause misinterpretation of sensory information, leading to delusions about the body [11]. This case reinforces the importance of thorough neuropsychiatric assessment when patients present with psychiatric symptoms in the context of neurological injury. It also supports the further need for accumulation of evidence linking left frontal and insular pathology to psychosis, and highlights the need for further research into how focal brain lesions can drive psychiatric presentations [12].

## Conclusions

This case illustrates the complex relationship between brain pathology and psychiatric symptoms, particularly when somatic symptoms and delusions occur on the same side as a cerebral hemorrhage. The patient’s psychotic symptoms largely subsided, but she continued to experience trauma-related anxiety, particularly a persistent fear of dying in her sleep. Although she continued taking her prescribed medications, she was lost to follow-up after discharge due to not attending her scheduled outpatient appointment. It emphasizes the need to consider neuroanatomical and neurobiological factors when diagnosing psychiatric conditions, highlighting the impact brain lesions can have on psychiatric functioning and contributing to a deeper understanding of how organic brain disorders manifest as psychiatric symptoms.

Additionally, the case underscores the importance of a comprehensive, multidisciplinary approach to diagnosis and treatment. To translate this understanding into clinical practice, clinicians should consider establishing imaging thresholds for patients presenting with lateralized somatic symptoms and unexplained psychosis, incorporating routine psychiatric screening within neurology settings, and fostering stronger collaboration between neurologists and psychiatrists. Further research is needed to elucidate the role of organic brain lesions, such as aneurysms and intracranial hemorrhages, in the development of psychiatric symptoms. A deeper understanding of these associations could refine differential diagnoses, improve diagnostic accuracy, and help clinicians identify early somatic indicators of untreated aneurysms, facilitating timely intervention. Bridging the gap between neurobiology, neuroanatomy, and psychosis could offer valuable insights, expanding diagnostic pathways for clinicians.
